# 4D live tracing reveals distinct movement trajectories of meiotic chromosomes

**DOI:** 10.1093/lifemedi/lnae038

**Published:** 2024-11-13

**Authors:** Peng Xie, Shiqi Zhu, Jin Zhang, Xinrui Wang, Xu Jiang, Feng Xiong, Linjin Chen, Ke Fang, Yuanhui Ji, Beihong Zheng, Lincui Da, Hua Cao, Yan Sun, Zhuojuan Luo, Chengqi Lin

**Affiliations:** School of Biological Science and Medical Engineering, Southeast University, Nanjing 211102, China; Co-innovation Center of Neuroregeneration, Nantong University, Nantong 226001, China; School of Life Science and Technology, Southeast University, Nanjing 210096, China; School of Life Science and Technology, Southeast University, Nanjing 210096, China; Medical Research Center, Fujian Maternity and Child Health Hospital, College of Clinical Medicine for Obstetrics & Gynecology and Pediatrics, Fujian Medical University, Fuzhou 350004, China; School of Life Science and Technology, Southeast University, Nanjing 210096, China; School of Biological Science and Medical Engineering, Southeast University, Nanjing 211102, China; Medical Research Center, Fujian Maternity and Child Health Hospital, College of Clinical Medicine for Obstetrics & Gynecology and Pediatrics, Fujian Medical University, Fuzhou 350004, China; School of Life Science and Technology, Southeast University, Nanjing 210096, China; Jiangsu Province Hi-Tech Key Laboratory for Biomedical Research, School of Chemistry and Chemical Engineering, Southeast University, Nanjing 211102, China; Center of Reproductive Medicine, Fujian Maternity and Child Health Hospital, Fuzhou 350001, China; Center of Reproductive Medicine, Fujian Maternity and Child Health Hospital, Fuzhou 350001, China; Medical Research Center, Fujian Maternity and Child Health Hospital, College of Clinical Medicine for Obstetrics & Gynecology and Pediatrics, Fujian Medical University, Fuzhou 350004, China; Center of Reproductive Medicine, Fujian Maternity and Child Health Hospital, Fuzhou 350001, China; Co-innovation Center of Neuroregeneration, Nantong University, Nantong 226001, China; School of Life Science and Technology, Southeast University, Nanjing 210096, China; Shenzhen Research Institute, Southeast University, Shenzhen 518057, China; Co-innovation Center of Neuroregeneration, Nantong University, Nantong 226001, China; School of Life Science and Technology, Southeast University, Nanjing 210096, China; Center of Reproductive Medicine, Fujian Maternity and Child Health Hospital, Fuzhou 350001, China; Shenzhen Research Institute, Southeast University, Shenzhen 518057, China

**Keywords:** chromosome segregation, chromosome movement trajectory, live imaging, deep learning

## Abstract

Proper chromosome alignment at the spindle equator is a prerequisite for accurate chromosome segregation during cell division. However, the chromosome movement trajectories prior to alignment remain elusive. Here, we established a 4D imaging analysis framework to visualize chromosome dynamics and develop a deep-learning model for chromosome movement trajectory classification. Our data reveal that chromosomes follow at least three distinct movement trajectories (retracing, congressing, and quasi-static) to arrive at the equator. We further revealed the distinct roles of multiple kinesin superfamily proteins (KIFs) in coordinating and maintaining the chromosome movement trajectories. In summary, we have presented an efficient and unbiased approach to studying chromosome dynamics during cell division, thereby uncovering a variety of chromosome movement trajectories that precede alignment.

## Introduction

Chromosome segregation during meiosis is a fundamental biological process essential for the accurate transmission of genetic information from parents to offspring. Aberrations in meiotic chromosome segregation can lead to severe consequences, including spontaneous miscarriage and a range of congenital disorders, such as trisomies of chromosome 21, 13, or 18 [1]. The spindle, a microtubule-based cellular structure, orchestrates the movement, alignment, and segregation of chromosomes, and its precision in managing this process is of paramount importance in safeguarding genome integrity across generations [2].

Prior to segregation, it is vital that chromosomes are accurately aligned at the spindle equator. This alignment process requires dynamic movement of chromosomes driven by the spindle’s pulling force during prophase, a crucial phase preceding alignment. Chromosomes undergo dramatic and dynamic back-and-forth movements during prophase that ultimately lead to their alignment at the equator. This pattern is distinct from that observed in anaphase, where chromosomes adopt a bipolar orientation. Previous studies have emphasized the significance of chromosome movement mediated by spindle in achieving successful alignment [3]. However, our understanding of the specific trajectories of chromosome movement along the spindle during meiotic prophase remains limited.

Kinesin superfamily proteins (KIFs) utilize the energy from ATP hydrolysis to move along the microtubule tracks, pivotal in coordinating spindle organization and dynamics during cell division. This ensures the precise alignment and equal distribution of chromosomes to daughter cells. The various KIFs fulfill distinct roles in orchestrating mitosis and meiosis, including managing microtubule dynamics, ensuring kinetochore alignment, and facilitating cytokinesis [4, 5]. For instance, KIF11, a classic motor protein which facilitates bipolar spindle formation by cross-linking and separating antiparallel microtubules, thereby generating the forces required to establish and sustain the spindle’s bipolar structure [6]. KIF2A, a spindle pole-localized kinesin, is involved in microtubule destabilization [7, 8]. CENPE, along with KIF4A and KIF18A, plays a critical role in kinetochore alignment by mediating the attachment of chromosomes to spindle microtubules and ensuring proper tension and orientation at the kinetochores [9, 10]. Nevertheless, whether and how KIFs regulate chromosome trajectory during cell division remains elusive.

To gain a deeper understanding of chromosome dynamics during meiosis, we conducted a comprehensive study using living mouse oocytes. By employing full-frame 4D spindle and single chromosome tracing techniques, we captured the dynamic nature of chromosome movement during alignment in prophase I of meiosis. This approach enabled us to observe and analyze the trajectories of individual chromosomes real time. Our study uncovered dynamic mobility changes in chromosomes during prophase I of meiosis, specifically following three unique trajectories: retracing, congressing, and quasi-static. Deficiencies in KIF11 and KIF2A significantly but differently impact chromosome trajectories. KIF11 depletion slows or stalls chromosome movement, while KIF2A depletion results in rapid but chaotic chromosome movements. In addition, depletion of KIF18A or KIF4A tends to affect the retracing trajectories. In conclusion, our study sheds new light on the intricate dynamics of chromosome movement during meiosis, highlighting the pivotal role of KIFs in orchestrating proper chromosome trajectories preceding chromosome alignments.

## Results

### 4D tracing of meiotic chromosomes and spindle in living oocytes

**Figure 1.**
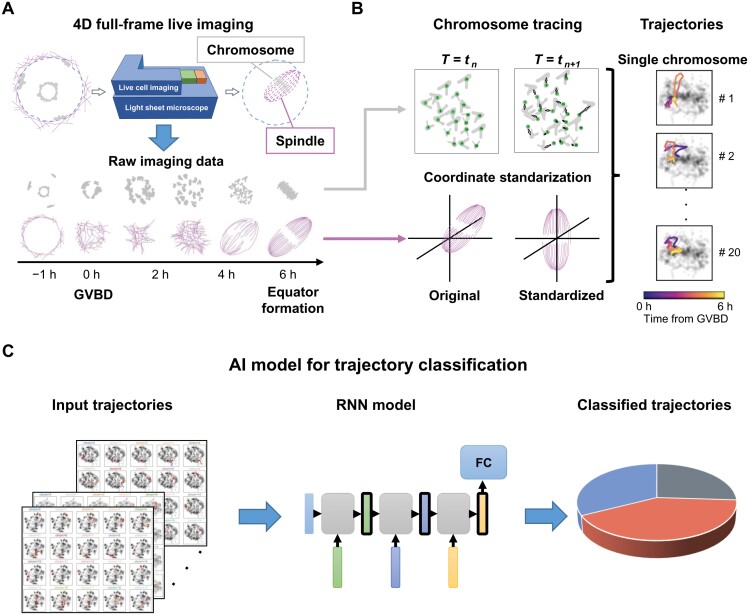
**4D chromosome tracing and trajectory analysis in mouse meiosis.** (A) 4D full-frame live imaging of chromosomes and spindles in mouse oocytes, from the onset of GVBD to the formation of the metaphase equator during MI using light-sheet microscope. (B) Examples of chromosome tracing between two adjacent time points. Arrows showing the displacement of chromosomes from time (*T*) *t_n_* to *t*_*n*+1_. Adjustment of image locations: each time point is re-located so that the center of chromosomes matches the center of image (upper left). In addition, the shape of the spindle at each time point is estimated by ellipsoid fitting (lower left). Reconstruction of signal chromosome movement trajectories based on the tracing results (right panel). (C) Classification of the different types of trajectory patterns by a RNN model.

To monitor the spatiotemporal dynamics of chromosomes and spindle till chromosome alignment, we performed 4D full-frame live imaging in mouse oocytes by using fast-acquisition and low photo-toxicity light-sheet microscopy. Spindle and chromosomes were respectively labeled with α-Tubulin-eGFP and histone H2B-mCherry, and imaged from germinal vesicle breakdown (GVBD), the onset of meiosis I (MI), to chromosome alignment at the MI spindle equator (Fig. 1A and Videos S1 and S2). We estimated the shapes of the spindles through ellipsoid fitting based on the Tubulin channel images. The imaged chromosomes were distinguishable from each other, allowing us to trace single chromosome trajectories via the 3D Visualization-Assisted Analysis Vaa3D software package [11]. To avoid interference from sample shifting and rotation, cross-time-point image alignment was performed according to the centers of chromosomes (Fig. 1B). In addition, we trained a Recurrent Neural Network (RNN) model, which is widely used in machine learning to process sequential data [12, 13], to categorize the observed trajectories (Fig. 1C).

### Speed dynamics of chromosome movement during prophase I of meiosis

**Figure 2.**
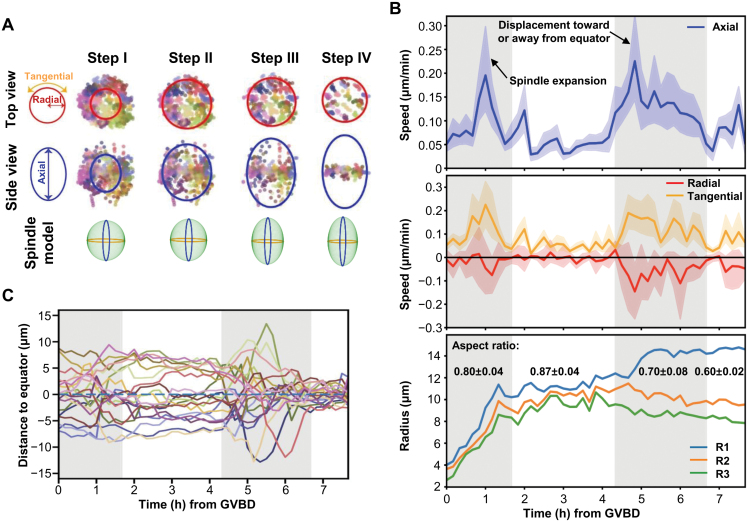
**Spindle and chromosome dynamics prior to the formation of metaphase equator during MI.** (A) Top-/side-views showing chromosome locations and spindle sizes in four steps. Arrows indicating the direction of chromosome movement, relative to spindle. (B) Chromosome speed estimated at each time point. Solid lines representing the average axial speed (upper panel), the radial and tangential speed (middle panel), and the shades representing 95% confidence intervals. The radius of spindle, R1 to R3 corresponding to the longest to the shortest radius (lower panel). (C) The distance of chromosomes to the equator plane across the four steps. Each colored curve corresponding to one chromosome.

Our full-frame chromosome/spindle imaging and single chromosome tracing framework offers us a unique advantage to perform unbiased quantitative analysis in the complete process of prophase I of meiosis. We applied the framework to analyze the chromosome movement in different directions relative to the forming spindle, including rotational, centripetal, and axial movements, and to measure the respective tangential, radial, and axial speeds (Fig. 2A and 2B). The observed chromosomal dynamic patterns supported a model wherein the process from GVBD to the MI chromosome alignment can be divided into four steps (Fig. 2A). Consistent with the previous report [3], at the initial step (Step I) soon after GVBD, the microtubule ball quickly formed and expanded; chromosomes moved outward (Fig. 2A). A sharp increase in chromosome movement speed (about 0.17 μm/min) was observed along the future spindle pole-to-pole axis, higher than that in the radial or tangential speed (Fig. 2B). At the second step (Step II), chromosomes were located near the outer surface of the microtubule ball and distributed evenly along the axis direction (Figs. 2A and S1A). Chromosomes displayed low axial and tangential speed, and negligible radial speed, indicating that their movement was restricted to the surface of the spindle (Fig. 2B).

A drastic increase in axial speed was observed at the onset of Step III, with chromosomes maintaining an average speed of 0.18 μm/min throughout this step for over an hour (Fig. 2B, upper panel). This axial speed substantially exceeded the estimated speed, calculated as distance divided by time (~0.04 µm/min), necessary for chromosomes to move directly to the equator in a unidirectional manner. The accelerated speed could be due to either rapid local oscillations or extensive traveling. Analysis of individual chromosome trajectories supported the above hypothesis, indicating that chromosomes might cross the equator multiple times before achieving stable alignment (Fig. 2C; Fig. S1B #1, #5, and #13). Step III also showed the highest speed in both the radial and tangential directions. The maximum radial speed of chromosome movement toward the spindle axis reached 0.10 μm/min (Fig. 2B, middle panel). Top views confirmed the rapid concentration of chromosomes from the periphery of the spindle (Fig. S1C). Meanwhile, according to the ellipsoid model of the spindle, the bi-oriented spindle elongated by 2 µm while the radius of the midplane decreased by 2 µm (Fig. 2A and 2B). As Step IV approached metaphase, the spindle continued to elongate while chromosome movement speeds were generally steady yet slow, ultimately resulting in the equatorial alignment of chromosomes (Fig. 2A and 2B).

### Non-unified chromosome movement trajectories during prophase I of meiosis

**Figure 3.**
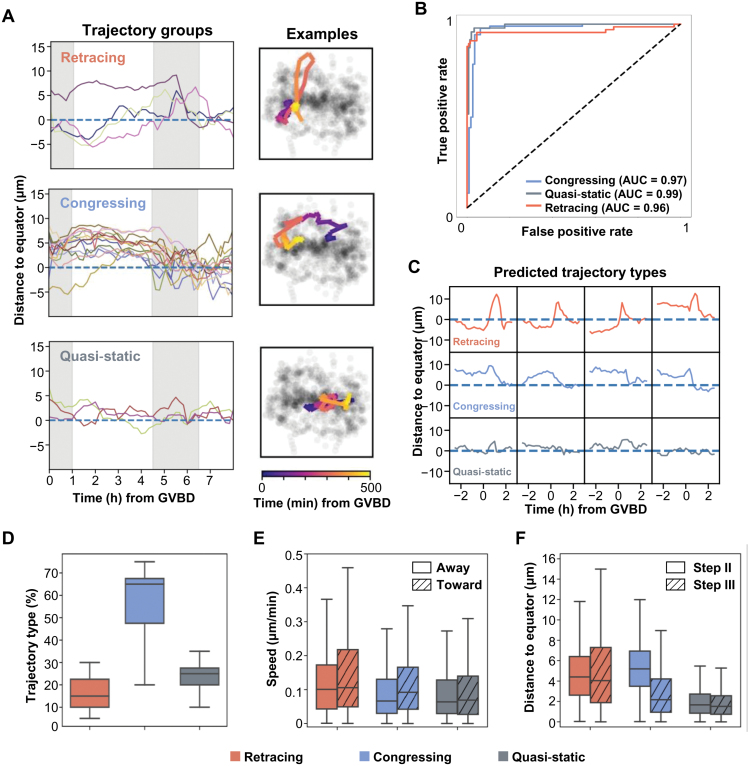
**Classification of chromosomal trajectories during meiosis.** (A) Classification of chromosome movement trajectories into three groups: colored curves for each group (left panel); side-views of trajectories of representative chromosomes (colored curves); semi-transparent dots showing all chromosomes across the four steps (right panel). (B) Receiver operating characteristic curves of the RNN trajectory classification model. Colors of curves corresponding to the three trajectory types. (C) Examples of trajectories and their predicted types. Curves are aligned by the start of meiosis Step III. (D–F) Boxplots of the ratio of the three trajectories (D), the chromosomal speed during Step III (E) and the chromosomal distance to equator planes during Step II and III (F). Whiskers showing the 1.5 interquartile ranges (IQR) past the low and high quantiles. Speed is estimated for movement toward or away from the equator plane (E).

It has been previously reported that chromosomes form an intermediate configuration, known as the prometaphase belt, prior to congressing toward the equator [3]. However, our full-frame spindle and single chromosome trajectory analyses revealed a more complex scenario. Specifically, not all of the 20 chromosomes followed the direct prometaphase belt-to-equator congressing trajectory (Fig. 3A, middle panel). A subset of chromosomes adopted a quasi-static trajectory. These chromosomes exhibited minimal movement, with an average displacement of less than 1.5 μm from the spindle midplane throughout the entire period from GVBD to the end of metaphase.

Intriguingly, we discovered that another subset of the chromosomes initially traveled ~12 μm away from the spindle midplane, nearing the spindle’s pole regions, before commencing their direct movement toward the equator. This movement pattern gave rise to retracing trajectories (Fig. 3A, upper panel and Fig. S2). During the retracing phase at Step III, this subset of chromosomes moved parallel to the spindle’s pole-to-pole axis at a significantly accelerated rate, reaching a maximum speed of 0.30 μm/min. Remarkably, the observation that some of the chromosomes followed these accelerated retracing trajectories at Step III was consistently observed in all examined oocytes that progressed through MI with normal timing. Therefore, there exist at least three distinct chromosomal trajectories, namely retracting, congressing, and quasi-static, before the chromosomes aligning at the equator.

We then trained a RNN model to classify the trajectories. The RNN model demonstrated excellent ability to distinguish the retracing, congressing, and quasi-static trajectories (Fig. 3B and 3C, AUC > 0.96), in turn confirming the distinction among the trajectory types. We further computed a range of metrics, including accuracy, sensitivity, and specificity to depict the classification framework (Table S1). We classified 200 chromosomal trajectories from 10 wild-type MI oocytes. Notably, we observed the heterogeneity in trajectory type composition among individual oocytes, which could be associated with the developmental potential of the oocytes (Fig. 3D). According to data averaged across oocytes, ~15% of the chromosomes followed the retracing trajectory, about 65% complied with the congressing trajectory, while the remaining chromosomes were quasi-static (Fig. 3D). Retracing chromosomes exhibited higher speeds compared to congressing and quasi-static ones (one-sided Mann–Whitney *U* test, adjusted *P*-value < 0.05, Fig. 3E). In addition, the retracing group showed a statistically significant greater distance to the equator plate in Step III than the congressing and quasi-static groups (Fig. 3F).

### Roles of KIFs in orchestrating and maintaining proper chromosome trajectories

**Figure 4.**
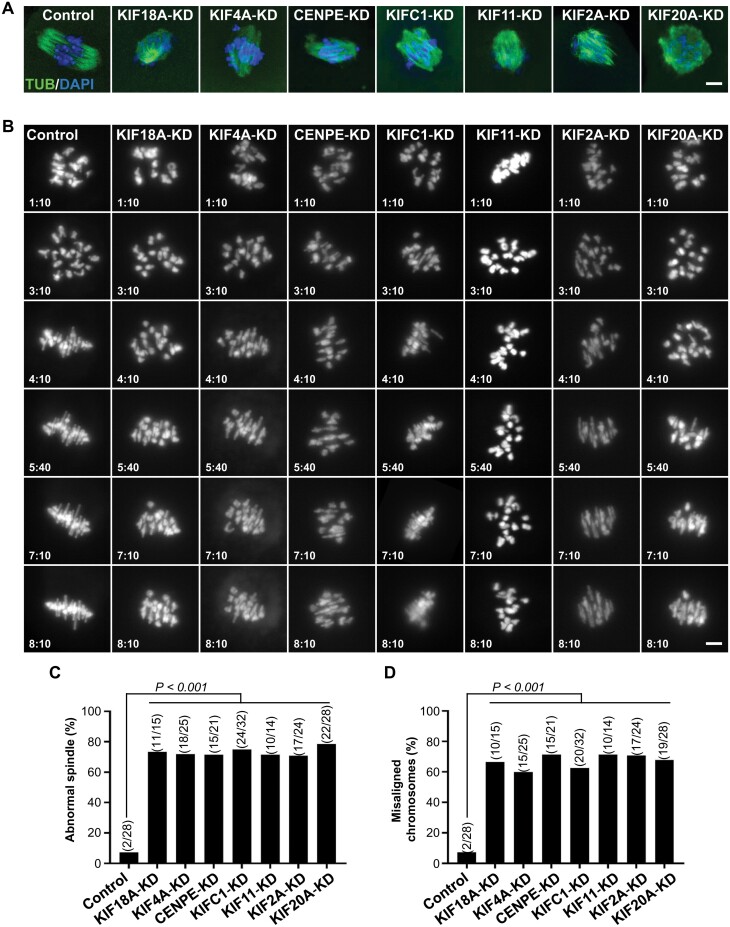
**Abnormalities of spindles and chromosome alignment after KIF-KD.** (A) Immunofluorescence images of the MI spindles in control, KIF18A-KD, KIF4A-KD, CENPE-KD, KIFC1-KD, KIF11-KD, KIF2A-KD, or KIF20A-KD mouse oocytes. Scale bars, 5 μm. (B) Snapshots of chromosome 4D imaging under control and each KIF-KD conditions. For each condition, representative maximum intensity projection snapshots are show for indicated time after GVBD. Scale bars, 5 μm. (C) Percentage of oocytes with abnormal spindle in control and KIF-KD mouse oocytes. (D) Percentage of oocytes with misaligned chromosomes in control and KIF-KD mouse oocytes. (C, D) Fisher's exact test, *P* < 0.001.

The existence of these distinct chromosomal trajectory patterns (retracing, congressing, and quasi-static) suggests the presence of multiple regulatory mechanisms controlling chromosome movement trajectory during the alignment process. Chromosome movement is primarily driven by microtubule force exerted by various KIFs. Previous studies have established the roles of KIF11, KIFC1, and KIF2A in managing microtubule dynamics, CENPE, KIF4A, and KIF18A in kinetochore alignment, and KIF20A in cytokinesis [4, 5, 14, 15]. To pinpoint which KIFs orchestrate these chromosomal trajectories, we undertook RNAi experiments to knockdown (KD) these specific KIFs. Immunostaining experiments and 4D imaging showed that KIF depletion resulted in abnormal spindles and ill-shaped chromosomal configuration, supporting the importance of those KIFs in spindle formation and chromosome alignment during meiosis (Fig. 4A–D; Videos S3–S10). Notably, the spindle morphologies observed upon depletion of various KIFs were consistent with previously published phenotypes and demonstrated remarkable diversity (Fig. 4A) [16–24].

**Figure 5.**
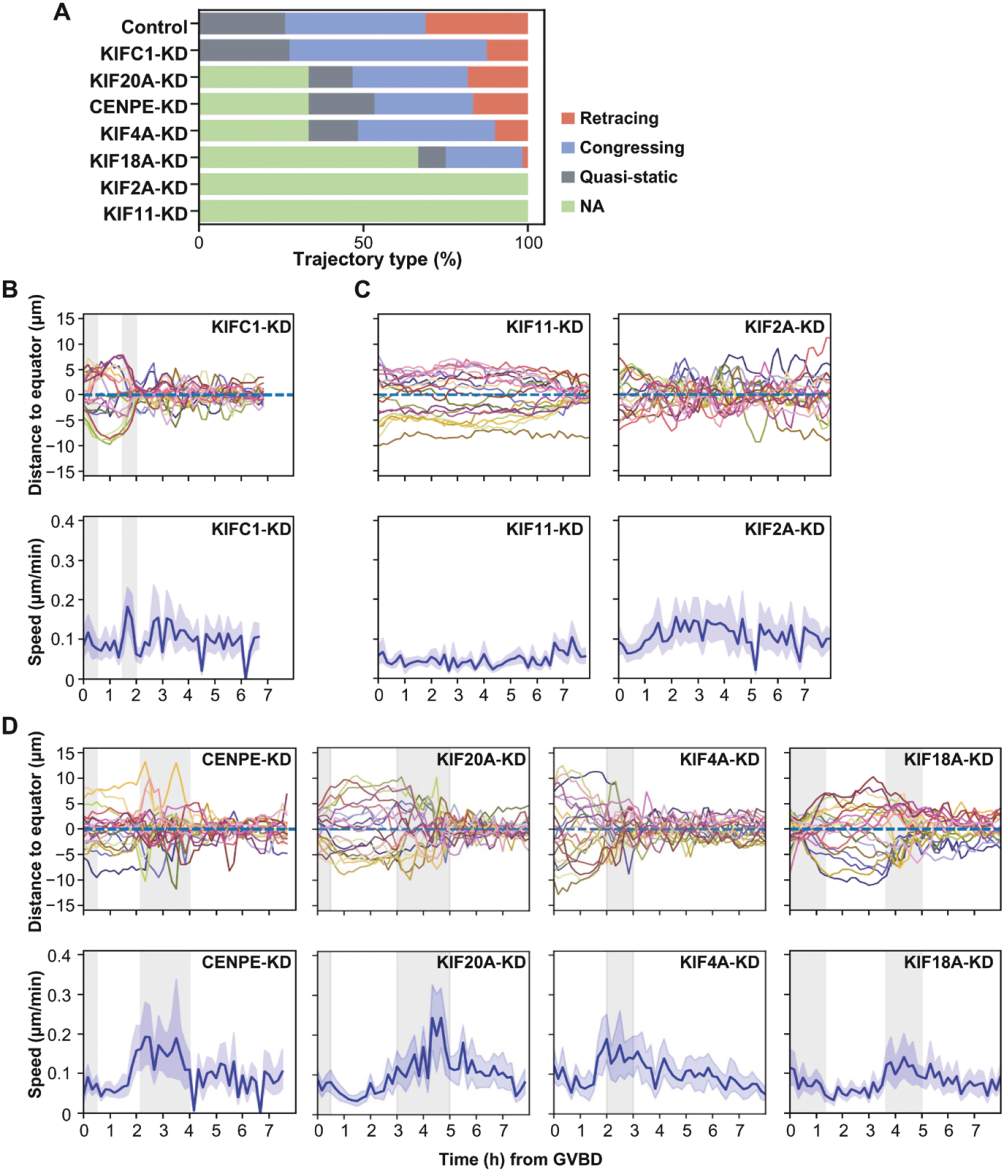
**Roles of KIFs in coordinating proper chromosome trajectories.** (A) Percentage of different trajectory types observed in control and KIF-KD mouse oocytes. Chromosomes from severely affected oocytes were manually labeled as NA (not applicable). (B–D) The distance of chromosomes to the equator plane after KIFC1-KD (B), KIF11-KD, KIF2A-KD (C), CENPE-KD, KIF20A-KD, KIF4A-KD, and KIF18A-KD (D) in mouse oocytes. Each colored curve corresponding to one chromosome (upper panel). Axial chromosome speed estimated at each time point after KIFC1-KD (B), KIF11-KD, KIF2A-KD (C), CENPE-KD, KIF20A-KD, KIF4A-KD, and KIF18A-KD (D) in mouse oocytes. Solid lines representing the average axial speed, and the shades representing 95% confidence intervals (lower panel).

We subsequently applied our analytical framework to assess the functional importance of these KIFs in shaping chromosome movement trajectories (Fig. 5A). The trajectory analysis revealed that the phenotypes arising from KIF depletion can be graded in terms of severity. For instance, KIFC1-KD belonged to the least severe category, where both the equator formation and the four steps were clearly identifiable (Fig. 5B). In the most severe cases, the equator configuration failed to form even 8 h after GVBD (Fig. S3). Both KIF11 and KIF2A, when depleted, exhibited phenotypes belonging to this severe category, causing complete disruption in chromosome trajectories and significant defects in chromosome alignment, albeit with distinct functional differences. Specifically, KIF11 depletion led to sluggish or even stationary chromosomes, whereas KIF2A depletion induced rapid and erratic chromosomal movements (Fig. 5C). In instances of mild severity, where equators were visible, we classified chromosomal trajectories using the RNN model. Results indicated that the ratio of retracing trajectories was notably lower in KIF18A-KD and KIF4A-KD oocytes compared to wild-type oocytes, while the quasi-static ratio remained largely unaffected (Fig. 5D).

## Discussion

**Figure 6.**
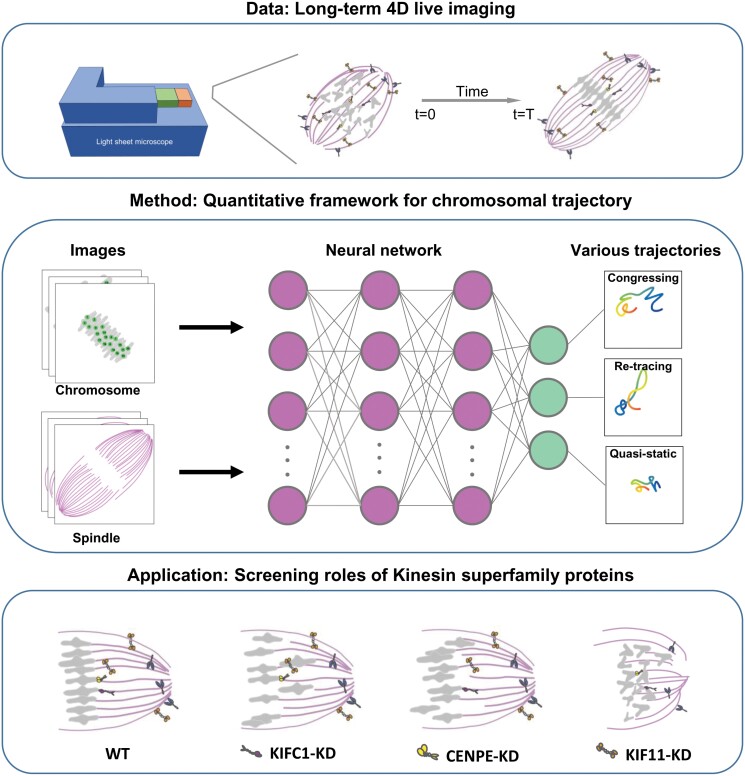
**Flowchart of the study and summary of the key findings.** The current study, utilizing long-term 4D live imaging coupled with a robust quantitative analysis framework, has unveiled intricate chromosome movement patterns during meiosis in mouse oocytes. By integrating machine learning models, we have achieved precise tracking and classification of chromosome trajectories throughout the meiotic process. Our results highlight three distinct patterns of chromosome movement: congressing, retracing, and quasi-static, thereby emphasizing the dynamics of chromosomes prior to their alignment.

This study, through long-term 4D live imaging and a quantitative analysis framework, has uncovered chromosome movement patterns during meiosis in mouse oocytes. This approach combines machine learning models, enabling precise tracking and classification of chromosome trajectories during meiosis. Our findings reveal three distinct patterns of chromosome movement—congressing, retracing, and quasi-static—underscoring the complexity of chromosome dynamics prior to alignment (Fig. 6).

Our study underscores the specific roles played by different KIFs in chromosome movement trajectories. Specifically, the depletion of KIF11 and KIF2A led to opposing effects on chromosome movement, with KIF11-KD resulting in slower or static chromosomes and KIF2A-KD causing faster and chaotic movements. This highlights the importance of balanced regulation of microtubule dynamics and force coordination along microtubule tracks for accurate chromosome segregation. In contrast, the minimal impact of KIFC1-KD on the chromosome movement trajectory aligns with previous findings indicating KIFC1 deficiency in human oocytes [25], suggesting that KIFC1 may not be absolutely essential for oocyte meiosis, despite its ability to stabilize the human meiotic spindle.

Moreover, we observed that certain KIFs, such as KIF18A and KIF4A, affect the ratio of retracing trajectories but not the quasi-static ratio, suggesting their involvement in regulating the initial poleward movement of chromosomes. In line with our findings, a recent study identified KIF18A and KIF4A as regulators of microtubule overlap and poleward flux, facilitating chromosome alignment [26]. Thus it is likely that the flux generated by forces within the microtubules overlaps could regulate chromosome alignment by influencing their movement trajectories.

## Research limitation

A limitation of our study is that we have not yet combined live cell chromosome labeling techniques with our tracing approach to determine if certain chromosomes exhibit specific movement trajectories. Recent studies using single cell karyotype sequencing have reported that chromosomes have varying probabilities of segregation errors [27]. By integrating live cell chromosome labeling with our tracking technology in future research, we may gain valuable insights into whether aberrations in trajectory patterns contribute to specific aneuploid errors.

## Methods

### Research ethics

All mouse experiments were approved by the Animal Care and Use Committee at Southeast University and performed in accordance with institutional guidelines and ethical standards (No. 20220105010).

### Mice

The mice were housed in cages under SPF conditions and had free access to water and food. Female ICR mice aged 3–6 weeks were used for the purpose of oocyte production for the microinjection experiments.

### Oocyte collection and culture

Female mice aged 3–6 weeks were injected with 5 IU of pregnant mare serum gonadotropin (PMSG, San-Sheng Pharmaceutical). Fully grown oocytes at the GV stage were collected 44–48 h after PMSG administration and released directly into M2 medium (Sigma) supplemented with 200 μM 3-isobutyl-1-metyl-xanthine (IBMX, Sigma). During handling and microinjection procedures, oocytes were cultured in M2 medium at 37°C, with the addition of 200 μM IBMX to arrest them at prophase I, if necessary. The oocytes were randomly selected for microinjection. To stimulate the meiotic maturation *in vitro*, the oocytes were washed out of IBMX, and cultured in drops of M16 medium (Sigma) under mineral oil (Sigma) in a 5% humidified CO_2_ incubator at 37°C. The oocytes were subsequently collected at various time points for further analysis.

### In vitro transcription and microinjections

Tubulin-eGFP and H2B-mCherry capped cRNAs were synthesized from linearized plasmids using the T7 mMessage mMachine kit (Ambion). The resulting transcripts were then poly-A tailed with the Poly(A) tailing kit (Ambion), followed by purification using the mEGAclear kit (Ambion). The purified cRNAs were dissolved in RNase-free water (Ambion) for further use. Typically, approximately 5–10 μL of mRNA, at a concentration of 500 ng/μL, was microinjected into the GV oocytes at room temperature, utilizing an Eppendorf micromanipulator mounted on an OLYMPUS inverted microscope. Following injection, the oocytes were cultured in M2 medium containing 0.2 mM IBMX at 37°C with 5% CO_2_ for a minimum of 3 h to enable protein expression. For KD experiments, the working concentration of siRNA was set at 25 μM. To promote the degradation of mRNA by siRNA, the microinjected oocytes were arrested at the GV stage for 16–20 h. After this arrest period, the oocytes were transferred into IBMX-free M16 medium to resume meiosis for subsequent experimental procedures.

The siRNA duplexes were designed based on mouse mRNA sequences and synthesized by GenePharma (Shanghai, China). Sequences of the sense strands are as follows:

siControl: 5ʹ-UUCUCCGAACGUGUCACGUTT-3ʹ;

siKIF11#1: 5ʹ-AGCAAAGAACATAATGAATAA-3ʹ;

siKIF11#2: 5ʹ-CACAGGAACTTTGCCAGTTAA-3ʹ;

siKIFC1#1: 5ʹ-TACACTGGGACTGGTCATAAT-3ʹ;

siKIFC1#2: 5ʹ-GTGAACAATATTTATTATGTA-3ʹ;

siCENPE#1: 5ʹ-CAAGGCTACAATGGTACTATA-3ʹ;

siCENPE#2: 5ʹ-AAGCATTGGGCTCGTGAATAA-3ʹ;

siKIF4A#1: 5ʹ-AAGAATTGGCTTGGAAATGAA-3ʹ;

siKIF4A#2: 5ʹ-TACGATGAAATACATGGTCAA-3ʹ;

siKIF2A#1: 5ʹ-AAGGAGTGCATCCGAGCCTTA-3ʹ;

siKIF2A#2: 5ʹ-CTGCTGGACCATTCCATCTTA-3ʹ;

siKIF18A#1: 5ʹ-TGCATTGTAAATATTGTTTAA-3ʹ;

siKIF18A#2: 5ʹ-CAGATTTATTTGCGACAACAA-3ʹ;

siKIF20A#1: 5ʹ-CAGGAAGTTAAAGCTGAACTA-3ʹ;

siKIF20A#2: 5ʹ-CAGCTAGATGAAACAAGTCAA-3ʹ.

### Immunofluorescence

The oocytes were fixed in 4% paraformaldehyde in 1×PBS [pH 7.4] for 30 min. After permeabilization and blocking with PBS containing 0.5% Triton X-100 and 1 mg/mL BSA at room temperature, the oocytes were incubated with anti-α-Tubulin-FITC (1:200, Sigma) at 4°C overnight. After washing four times for 5 min each in PBS containing 0.1% Tween 20 and 0.01% Triton-X 100, oocytes were counterstained with Hoechst 33342 (10 mg/mL). Finally, the oocytes were observed under a confocal laser scanning microscope (Zeiss LSM 700). The acquired images were analyzed using Zen Light Edition.

### Live cell imaging

Imaging was conducted with the InVi SPIM inverted light-sheet microscope (Luxendo, Bruker Corporation, Germany). A 25-μm thin FEP film (Luxendo) was affixed to the upper surface of the sample holder using a biocompatible silicone adhesive (Silpuran 4200; Wacker), thus forming an imaging trough. Once fresh M16 medium was introduced, the sample was positioned in the imaging trough and allowed to settle overnight to minimize gel drift during the imaging process. The imaging chamber was situated within an incubator that maintained controlled environmental conditions and was submerged in water, ensuring that both the objective lenses and the base of the sample holder remained beneath the water surface.

The InVi SPIM is equipped with a Nikon CFI 10×/0.3NA water immersion lens for illumination and a Nikon CFI-75 25×/1.1NA water immersion lens for detection. For imaging microtubules, stacks of 80 images with 488 nm between planes were acquired simultaneously for eGFP signals at 10-min intervals with an exposure time of 50–100 ms. For imaging chromosomes, 80 images with 561 nm between planes were acquired simultaneously for mCherry signals at 10-min intervals with an exposure time of 50–100 ms. Three-dimensional image stacks were captured with a light-sheet thickness of 1 µm and a final magnification of 62.5×, which yielded a pixel size of 104 nm. The InVi SPIM is maintained under specific environmental conditions of 37°C, 5% CO_2_, and 95% humidity. To mitigate phototoxicity and prevent photo-bleaching, extensive testing has been conducted on various parameters, including laser power, exposure duration, and z-step size. The resulting optimal imaging conditions involve utilizing 488 nm and 561 nm lasers, an exposure time of 50–100 milliseconds per frame, and a z-spacing of 1 μm between frames. The oocytes imaging at least 8 h after the induction of the meiotic resumption.

### Single chromosome trajectory tracing

The moving trajectory of a single chromosome is traced by manual annotation of its location at each time frame. The first time point when individual chromosomes became distinguishable was determined as the beginning of tracing. A chromosome is annotated as a marker at the centers of signal blobs, using the open source software Vaa3D [11]. These markers were numbered to represent their identities. At each time point, the location of a specific chromosome is annotated by shifting its marker from the previous frame to current blob center. The identity of a chromosome is determined by comparison of local chromosomal conformation and morphologies between adjacent time frames. To avoid tracing errors, we calculated the distance of movement between adjacent time frames for each chromosome. Chromosomes were labeled for a second round of tracing, when its distance is above average plus twice standard deviation at corresponding time frame. Manual annotations were checked and independently repeated by multiple researchers.

To analyze chromosomal trajectories, we converted unit of marker coordinate as “μm” according to the resolution of microscopic imaging (*x*/*y* = 0.104 μm, *z* = 1 μm). To align the coordinates of different oocytes, we set the center of the chromosomes as the origin and the pole-to-pole axis *y*-axis. For each oocyte, we manually determined the first time point when equator formed and performed principal component analysis to identify the equator plane. Principal components (PC) 1–3 were set as *x*-, *y*-, and *z*-axis, respectively. For each time frame, all chromosomes were shifted so their center was at the origin and were rotated according to the PCs. The *z*-axis values were defined as distance to the equate plate. To examine the effect of whole nucleus rotation, we performed ellipse fit of spindles at each time point. Across a 200-min window, the standard deviation of long-axis orientation was 4.5°C, which correspond to an estimation error of 0.025 μm of the equate plate distance (assuming 8 μm chromosomal distance to the spindle center).

### Deep-learning model for trajectory classification

We implemented a recurrent neural network to classify the three types of chromosome trajectories: retracing, congressing, and quasi-static. Trajectories were aligned by the beginning time point of Step III (T0) and represented as sequences of distance to the equator planes (± 150 min from T0). Considering that the trajectory of chromosome movement has the characteristics of sequence data, we used the RNN model to extract and capture the key features of the trajectory, thereby achieving effective classification of the trajectory. The model consists of two parts, a GRU (gated recurrent unit)-based sequence encoding module and a multilayer perceptron (MLP) for classification module. GRU is one architecture of RNN, tracking states of sequences and outputting the sequence as a high-level feature representation. The GRU module consists of two simple GRU layers, which receives input sequences, and outputs the feature vector,  for next downstream networks. The MLP module takes the representation  as input, and produces the logits of three trajectory types. Finally, the “softmax” activation function transforms these logits into probabilities of trajectory predictions. The RNN classification model takes cross-entropy between prediction distribution and true-label distribution as model loss to train whole model weights. Chromosomal trajectories of wild-type oocytes were divided as training and testing data by 9:1 ratio. The trained RNN model was applied to trajectories of KIF-KD oocytes which with mild severity. The model is implemented using TensorFlow framework (version: 2.17.0). Parameters can be found in the source code.

### Statistical analysis

All percentages or values from at least 2–3 repeated experiments. Sample numbers and statistical tests are indicated with in the figures or figure legends. Data were analyzed by fisher exact test, provided by GraphPad Prism8 statistical software. The level of significance was accepted as *P*-value < 0.05.

## Supplementary Material

lnae038_suppl_Supplementary_Figures_S1-S3

lnae038_suppl_Supplementary_Table_S1

lnae038_suppl_Supplementary_Videos_S1-S10

## Data Availability

All data are described in the main text or supplementary materials. Source code for the deep-learning model is available via Github and Zenodo.
