# Supplementary material for: 4D live tracing reveals distinct movement trajectories of meiotic chromosomes
Source: Life Med. 2024 Nov 13;3(6):lnae038. doi: 10.1093/lifemedi/lnae038 (PMC11748274; doi:10.1093/lifemedi/lnae038)
Supplement: lnae038_suppl_Supplementary_Figures_S1-S3 [file lnae038_suppl_supplementary_figures_s1-s3.docx]

**4D live tracing reveals distinct movement trajectories of meiotic chromosomes**

Peng Xie^1,2,†^, Shiqi Zhu^3,†^, Jin Zhang^3,†^, Xinrui Wang^4,†^, Xu Jiang^3^, Feng Xiong^1^, Linjin Chen^4^, Ke Fang^3^, Yuanhui Ji^5^, Beihong Zheng^6^, Lincui Da^6^, Hua Cao^4^, Yan Sun^6,*^, Zhuojuan Luo^2,3,7,*^, Chengqi Lin^2,3,6,7,*^

^1^School of Biological Science and Medical Engineering, Southeast University, Nanjing 211102, China

^2^Co-innovation Center of Neuroregeneration, Nantong University, Nantong 226001, China

^3^School of Life Science and Technology, Southeast University, Nanjing 210096, China

^4^Medical Research Center, Fujian Maternity and Child Health Hospital, College of Clinical Medicine for Obstetrics & Gynecology and Pediatrics, Fujian Medical University, Fuzhou 350004, China

^5^Jiangsu Province Hi-Tech Key Laboratory for Biomedical Research, School of Chemistry and Chemical Engineering, Southeast University, Nanjing 211102, China

^6^Center of Reproductive Medicine, Fujian Maternity and Child Health Hospital, Fuzhou 350001, China

^7^Shenzhen Research Institute, Southeast University, Shenzhen 518057, China

^†^These authors contributed equally to this work.

*Correspondence: [sunyan62@163.com](mailto:sunyan62@163.com) (Y.S.), [zjluo@seu.edu.cn](mailto:zjluo@seu.edu.cn) (Z.L.), [cqlin@seu.edu.cn](mailto:cqlin@seu.edu.cn) (C.L.)

**
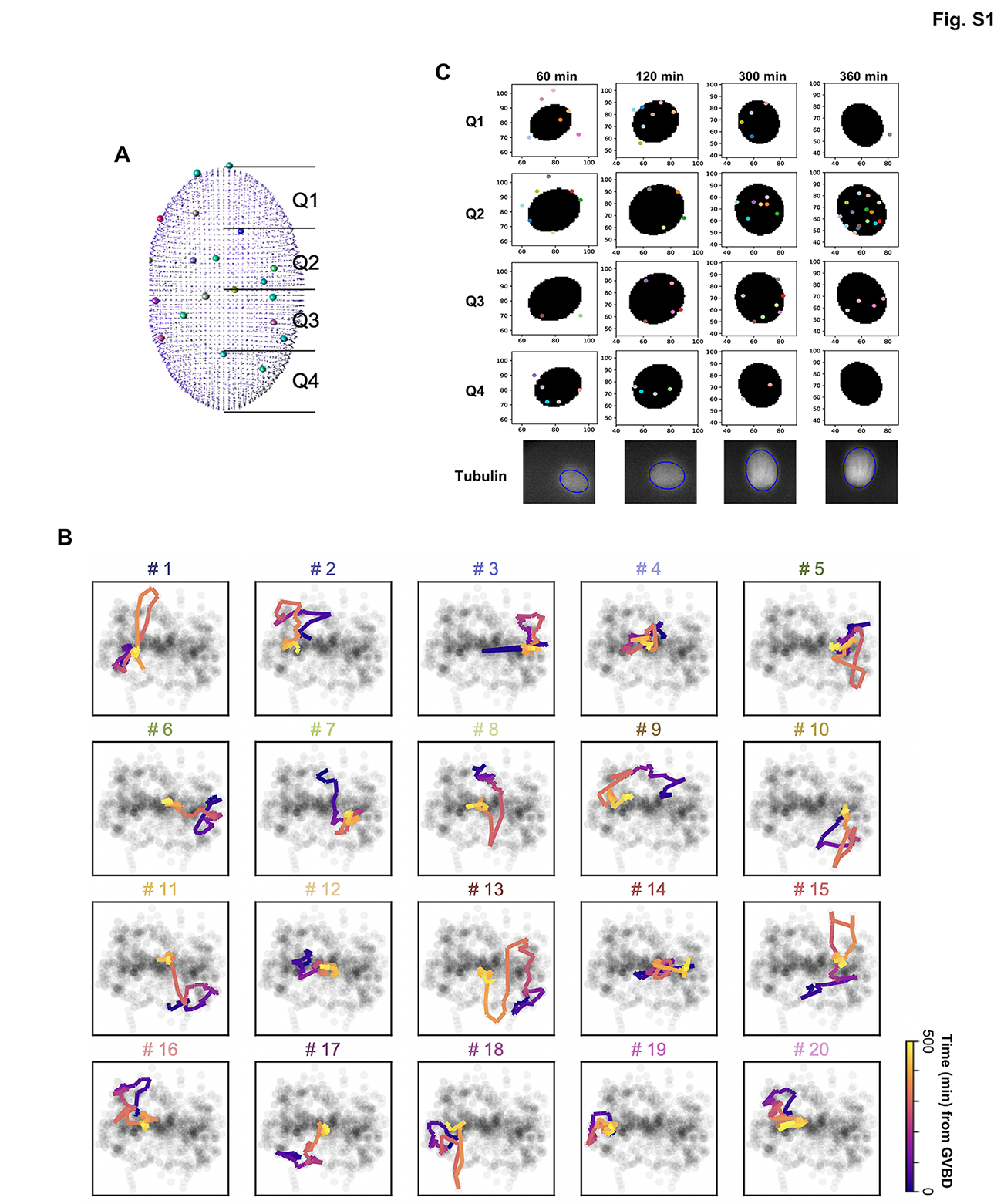
**

**Figure S1. Chromosome position distribution and side views of trajectories.**

(A) Side-views for chromosome position along the axial direction. The spindle is modeled as an ellipsoid, which is divided into four quarters (Q1–Q4). (B) Chromosome distribution in each spindle quarter. The chromosomes are assigned to each quarter and plotted in the top-view plane. The maximum intensity projection (MIP) of the spindle is shown as dark shade. The bottom panel showing the Tubulin (spindle) channel images with the blue lines of ellipsoid estimation. Samples of four representative time points are selected and shown in each column. (C) Chromosomal trajectories shown as side views. Trajectories are color-coded by the time points between 0 to 500 min after GVBD. Grey dots in the background representing locations of all chromosomes during the same time period.


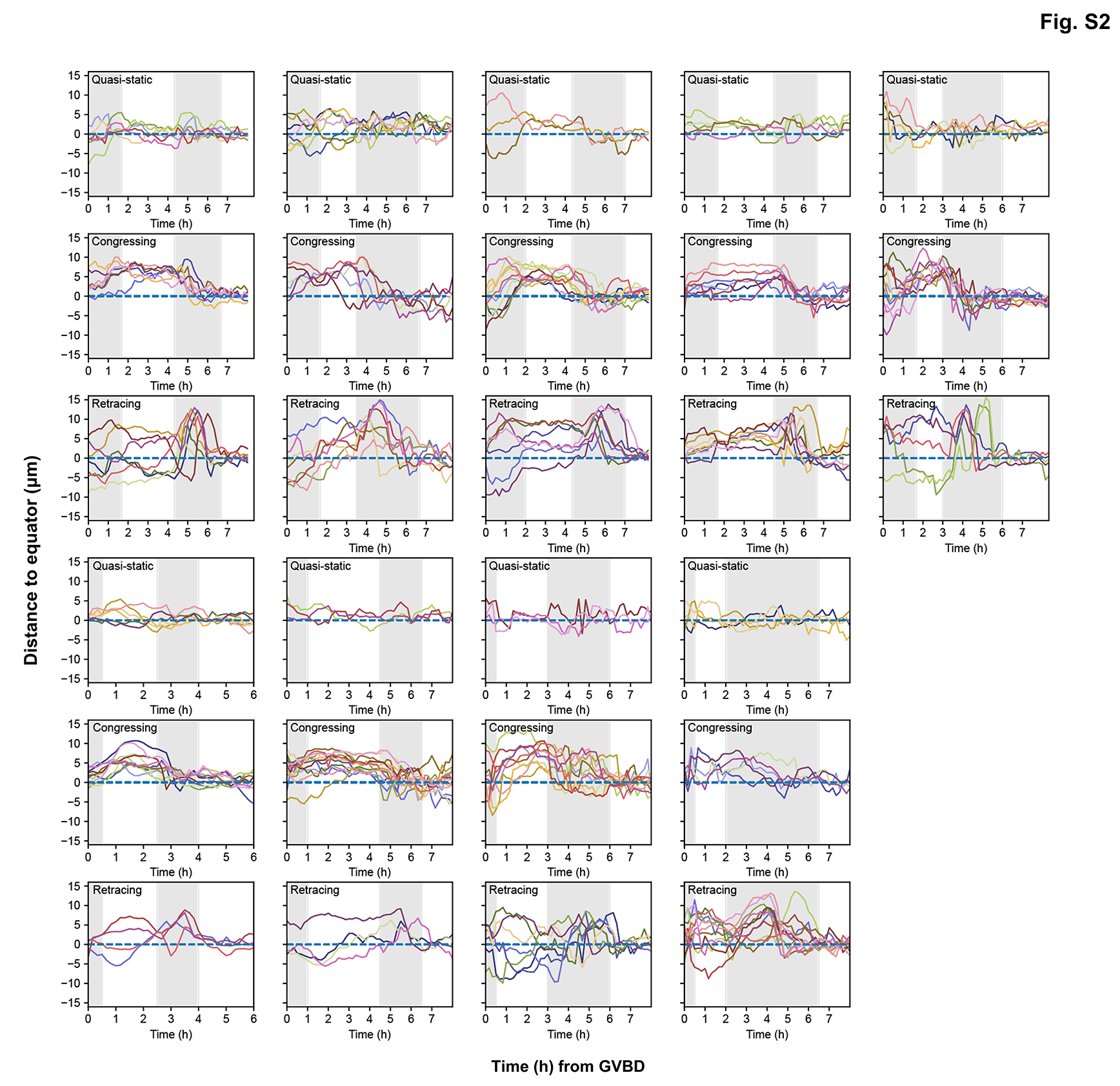


**Figure S2. Classification of chromosomal trajectories in wild-type oocytes.**

For each oocyte, trajectories are classified into three groups and shown in three subplots. Colored curves representing single chromosomes.


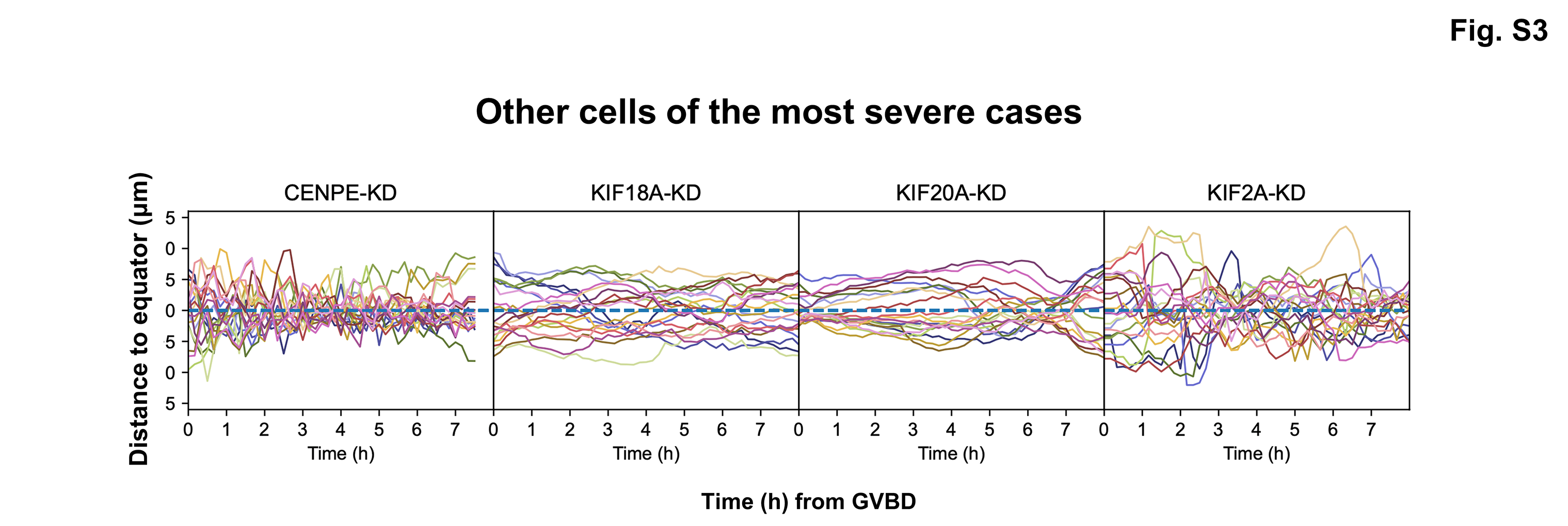


**Figure S3. Examples of chromosomal trajectories of the severely affected KIF-KD oocytes.**

Additional examples of Fig. 5C are shown. *Y*-axis shows the distance to the equator plane. Each colored curve matches one chromosome.
